# Investigation of local stimulation effects of embedding PGLA at Zusanli (ST36) acupoint in rats based on TRPV2 and TRPV4 ion channels

**DOI:** 10.3389/fnins.2024.1469142

**Published:** 2024-10-09

**Authors:** Xunrui Hou, Xin Liang, Yuwei Lu, Qian Zhang, Yujia Wang, Ming Xu, Yuheng Luo, Tongtao Fan, Yiyi Zhang, Tingting Ye, Kean Zhou, Jiahui Shi, Min Li, Lihong Li

**Affiliations:** ^1^Clinical Medical College of Acupuncture Moxibustion and Rehabilitation, Guangzhou University of Chinese Medicine, Guangzhou, China; ^2^Affiliated Hospital of Guizhou Medical University, Guiyang, China; ^3^Guizhou Medical University, Guiyang, China; ^4^The Affiliated TCM Hospital of Guangzhou Medical University, Guangzhou, China; ^5^Weihai Hospital of Traditional Chinese Medicine, Affiliated Hospital of Shandong University of Traditional Chinese Medicine, Weihai, China

**Keywords:** Acupoint Catgut Embedding, mechanosensitive TRPV channel, Zusanli (ST36), local stimulation, Ca^2+^ signaling

## Abstract

**Introduction:**

Acupoint Catgut Embedding (ACE) is an extended and developed form of traditional acupuncture that serves as a composite stimulation therapy for various diseases. However, its local stimulation effects on acupoints remain unclear. Acupuncture can activate mechanically sensitive calcium ion channels, TRPV2 and TRPV4, located on various cell membranes, promoting Ca^2+^ influx in acupoint tissues to exert effects. Whether ACE can form mechanical physical stimulation to regulate these channels and the related linkage effect requires validation.

**Methods:**

This study investigates the influence of TRPV2 and TRPV4 ion channels on the local stimulation effects of ACE by embedding PGLA suture at the Zusanli (ST36) acupoint in rats and using TRPV2 and TRPV4 inhibitors. Flow cytometry, immunofluorescence, Western blot, and Real-time quantitative PCR were employed to detect intracellular Ca^2+^ fluorescence intensity, the expression of macrophage (Mac) CD68 and mast cell (MC) tryptase, as well as the protein and mRNA expression of TRPV2 and TRPV4 in acupoint tissues after PGLA embedding.

**Results:**

The results indicate that ACE using PGLA suture significantly increases the mRNA and protein expression of TRPV2 and TRPV4, Ca^2+^ fluorescence intensity, and the expression of Mac CD68 and MC tryptase in acupoint tissues, with these effects diminishing over time. The increasing trends are reduced after using inhibitors, particularly when both inhibitors are used simultaneously. Furthermore, correlation analysis shows that embedding PGLA suture at the ST36 acupoint regulates Mac and MC functions through Ca2+ signaling involving not only TRPV2 and TRPV4 but multiple pathways.

**Discussion:**

These results suggest that embedding PGLA suture at the ST36 acupoint generates mechanical physical stimulation and regulates TRPV2 and TRPV4 ion channels, which couple with Ca^2+^ signaling to form a linkage effect that gradually weakens over time. This provides new reference data for further studies on the stimulation effects and clinical promotion of ACE.

## 1 Introduction

Acupoint Catgut Embedding (ACE) originates from the traditional acupuncture theory of “retaining needles,” utilizing absorbable sutures to provide continuous stimulation to acupoints for disease prevention and treatment (Guan et al., 2009; Huo et al., 2017). Due to its minimal invasiveness, simple operation, long-lasting stimulation, and low frequency of visits (Guo M. et al., 2022; Zhang X. H. et al., 2023), it has been widely used in clinical practice to treat various systemic diseases (Cheng et al., 2022b). Concurrently, research on the mechanisms of ACE has gradually increased (Wei et al., 2019). However, previous mechanism studies have mainly focused on therapeutic effects or distal acupoint effects (Huo et al., 2017; Teng et al., 2022; Duan et al., 2021), with a need for in-depth research on recognized target points (Huo et al., 2017; Wei et al., 2019). Local acupoints are the initial response sites for acupuncture effects (Chen et al., 2013; Fu et al., 2023) and a common foundation for mechanism research (Li et al., 2015). Although acupoint stimulation effects are involved in various acupuncture methods (Min et al., 2015; Chen T. et al., 2016; Lowe, 2017; Chen et al., 2021), basic research on ACE in this area remains scarce. Therefore, exploring the stimulation effects formed by ACE-induced changes in the local microenvironment of acupoints provides an objective scientific basis for its promotion.

Macrophages (Macs) and mast cells (MCs) in the connective tissue of acupoint areas are generally considered to participate in initiating local stimulation effects (Fu et al., 2023; Jung and Lushniak, 2017). Acupuncture stimulation at acupoints can activate these two immune cells locally, transmitting stimulation signals (Wu et al., 2015; Yan et al., 2020; Yu et al., 2022). Previous research by our team found dynamic changes in the functional state of Macs and MCs in acupoint areas following ACE at the ST36 acupoint in rats. Besides the transient needle stimulation by embedding, the suture material as a foreign body causing a local immune inflammatory response is considered one of the stimulation effects post-ACE (Zhang Q. et al., 2023; Wang et al., 2023). However, as a composite stimulation therapy developed from traditional acupuncture, other local stimulation effects of ACE remain to be further studied (Wei et al., 2019; Xing et al., 2019).

It is known that in the transient receptor potential vanilloid (TRPV) family, TRPV2 and TRPV4 are mechanically sensitive calcium ion (Ca^2+^) channels (Shibasaki, 2016; Liedtke, 2005). When these channel proteins on different cell membranes perceive mechanical stimulation, the channels are opened, causing transmembrane Ca^2+^ movement into the cells (i.e., Ca^2+^ influx), triggering intracellular signal transduction and cell function activation (Liedtke and Kim, 2005). Recent studies have shown that acupuncture, as a mechanical physical stimulation, can activate TRPV2 and TRPV4 channels at acupoints, promoting Ca^2+^ influx, converting physical stimulation into biological information, and thus exerting acupuncture effects (Huang et al., 2018; Luo et al., 2022). Based on the above, on the one hand, ACE originates from traditional acupuncture and replaces needles with sutures to produce sustained stimulation at acupoints. Whether it can form mechanical physical stimulation in the acupoint area to regulate these two channels needs verification. On the other hand, as a composite stimulation therapy, in addition to causing Macs and MCs to participate in local immune inflammation response, it remains to be further verified whether ACE can couple the functions of these two immune cells through the TRPV2 and TRPV4 mechanosensitive channels on their cell membranes (Link et al., 2010; Chen et al., 2017; Michalick and Kuebler, 2020).

Therefore, this study aims to explore possible local mechanical physical and linkage stimulation effects of ACE by embedding poly(glycolide-co-lactide) (PGLA) sutures (Ke et al., 2020; Jain, 2000), known for their excellent mechanical properties and biocompatibility, at the ST36 acupoint in rats. By intervening with inhibitors of mechanically sensitive TRPV2 and TRPV4 channels, changes in TRPV mRNA and protein expression, intracellular Ca^2+^ fluorescence intensity, CD68 and tryptase expression in MACs and MCs in local tissues of acupoints were dynamically observed. Correlation analysis of the impact of intracellular Ca^2+^ fluorescence intensity on the CD68 and tryptase expression in Macs and MCs in acupoint areas provides new reference data for further studies on the mechanism of ACE stimulation effects and its clinical promotion.

## 2 Materials and methods

### 2.1 Experimental animals and grouping

**Figure 1:**
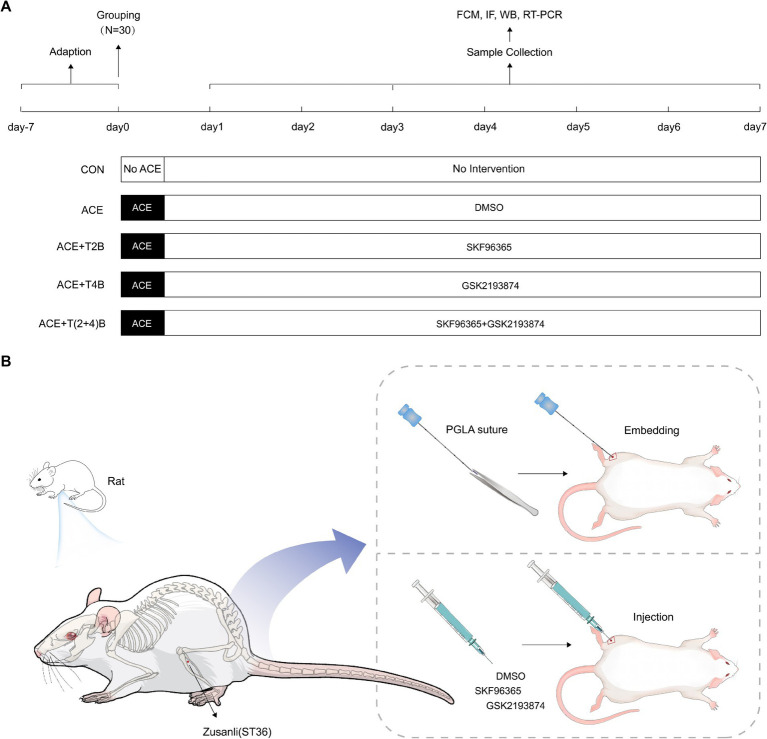
Experimental protocol. **(A)** Experimental procedures in this study. **(B)** Location of ST36 acupoint and interventions.

A total of 150 healthy male Sprague–Dawley (SD) rats (170-200 g, 8 weeks old) were purchased from Guangdong Vital River Laboratory Animal Technology Co., Ltd. (production license number: SCXK (Yue) 2022–0063) and housed in the SPF-level animal room of the Clinical Research Center, Affiliated Hospital of Guizhou Medical University. The housing conditions were maintained at a temperature of 22–24°C, humidity of 50–70%, with a 12-h light/dark cycle, and free access to food and water. After a one-week acclimatization period, the 150 rats were randomly divided into five groups (30 rats per group): Blank Control Group (CON), Embedding Group (ACE), Embedding + TRPV2 Inhibitor Group (ACE+T2B), Embedding + TRPV4 Inhibitor Group (ACE+T4B), and Embedding + TRPV (2 + 4) Inhibitor Group (ACE+T(2 + 4)B). Each group was further divided into three subgroups based on time points (1 day, 3 days, 7 days) with 10 rats in each subgroup (Figure 1A). The experimental protocol was approved by the Experimental Animal Ethics Committee of Guizhou Medical University (approval number: 2101330). During the experiment, the handling of animals strictly adhered to the “Guiding Opinions on Treating Experimental Animals Humanely” issued by the Ministry of Science and Technology of the People’s Republic of China in 2006. Before interventions, the rats were anesthetized with 3% sodium pentobarbital (P3761, Sigma, USA) administered intraperitoneally at a dose of 0.03 g/kg. The rats were then fixed in a prone position on a rat board with their limbs extended. The hair on the left hind limb was shaved, and the ST36 acupoint (located approximately 3 mm below the fibular head on the posterolateral side of the knee joint) was identified according to “Common Acupoint Names and Locations for Experimental Animals, Part 2: Rats” (China Association of Acupuncture and Moxibustion, 2021) and marked with a 1 cm × 1 cm square centered on this point (Figure 1B).

### 2.2 Intervention methods for each group

In the Embedding Group (ACE), rats underwent the ACE procedure at the marked ST36 acupoint on the left side once, and were injected daily with saline containing DMSO (the same amount as the ACE+T(2 + 4)B group). The embedding procedure was based on a method from previous studies by our research group (Wang et al., 2023): after local disinfection of the acupoint marking area, a PGLA suture (specification 2–0, 0.5 mm) (Shanghai Pudong Jinhui Medical Supplies Co., Ltd.) was placed into the cannula of a disposable embedding needle (0.9 mm × 75 mm, Taizhou Minga Medical Equipment Co., Ltd.). The acupoint was fixed with the thumb and forefinger of one hand, while the other hand held the needle, aligning the cannula with the skin at the center of the ST36 acupoint at a 90° angle. The needle was quickly inserted subcutaneously, then advanced slowly to a depth of about 7 mm, slightly twisted (two turns each to the left and right), and the needle core was pushed while withdrawing the needle to embed the suture into the acupoint. After needle withdrawal, the area was pressed with a disinfected cotton swab. Once the rats recovered from anesthesia, they were returned to their housing. In the (ACE+T2B) Group, (ACE+T4B) Group, and (ACE+T(2 + 4)B) Group, the rats underwent the same embedding procedure as described above. Additionally, at the center of the marked ST36 acupoint, they were injected with a mixed solution of SKF96365 (HY-100001, MCE, USA) (0.5 mg/kg) containing 0.5 mg/mL SKF96365 and 1% DMSO in saline, a mixed solution of GSK2193874 (HY-100720, MCE, USA) (0.5 mg/kg) containing 2.5 mg/mL GSK2193874, 5% DMSO, and 190 mg/mL sulfo-beta-cyclodextrin in saline, and a mixture of the above two solutions, respectively, once daily. In the Blank Control Group, rats received no other interventions besides the same handling and fixation method. All embedding operations and acupoint injections were performed by an experienced acupuncturist. To avoid cross-contamination, a single embedding needle and syringe were used for each rat’s acupoint only once. The acupoint area of the rats was observed daily for redness, swelling, or ulceration, and the acupoint area marking was reinforced (Figures 1A,B).

### 2.3 Tissue sampling

At the corresponding time points (1 day, 3 days, 7 days) post-intervention, tissue samples were collected from the five groups of rats. Following intraperitoneal anesthesia with 3% sodium pentobarbital (0.03 g/kg) and securing the rats on a board (the same fixation method as pre-intervention), tissue blocks (approximately 1 cm × 1 cm × 1 cm) from the marked ST36 acupoint region (including skin, subcutaneous tissue, and some muscle) were excised. Fresh tissue samples from five rats per group were randomly selected; half of each sample was fixed in 4% paraformaldehyde for further analysis, and the other half was used to prepare single-cell suspensions. The remaining five tissue samples were stored in a − 80°C freezer for future analysis.

### 2.4 Observation indicators and detection methods

#### 2.4.1 Flow cytometry

Subcutaneous fat was removed from the acupoint tissue, and the tissue was washed with Tyrode’s solution and cut into small pieces. Each gram of tissue was digested with 20 mL of digestion solution (collagenase type I and hyaluronidase, prepared in Hank’s solution with 20% fetal bovine serum) and incubated in a 37°C water bath for 4 h. The digested tissue was filtered through a 70 μm mesh sieve and centrifuged at 4°C, and the supernatant was discarded. Cells were washed twice with PBS and collected by centrifugation at 1500 rpm for 5 min. The cells were resuspended in serum-free DMEM medium, and 5 μmol/L Fluo 3-AM working solution (S1056, Shanghai Biyuntian Biotechnology Co., Ltd.) was added to the single-cell suspension. The cells were incubated in the dark at 37°C in a 5% CO_2_ incubator for 30 min, washed twice with calcium-free PBS, and resuspended to a final volume of 500 μL. The Ca^2+^ fluorescence intensity in the single-cell suspension was detected using a cytoFLEX flow cytometer (Beckman Coulter, USA).

#### 2.4.2 Immunofluorescence staining

The acupoint tissue fixed in 4% paraformaldehyde was dehydrated through a graded alcohol series, cleared in xylene, infiltrated with paraffin, and embedded. Sections of 4 μm thickness were prepared. The paraffin sections underwent processes including baking, dewaxing, antigen retrieval, and blocking with 10% normal goat serum for 30 min. The sections were incubated overnight at 4°C in a humid chamber with primary antibodies: anti-CD68 (1:200) (97778S, CST, USA) and anti-Mast Cell Tryptase (1:100) (bs-2572R, Bioss, USA). The next day, the primary antibodies were washed off, and the sections were incubated with secondary antibodies conjugated with fluorescent CY3 (goat anti-rabbit IgG, 1:100) (BA1032, Wuhan Boster Biological Technology, Ltd.) at 37°C for 1 h in a humid chamber. After washing off the secondary antibody, nuclei were stained with DAPI (5 min in the dark), excess DAPI was washed off, and the sections were mounted with antifade mounting medium. Images were observed and collected using a fluorescence microscope, with three fields of view per section at 400x magnification. Optical density analysis was performed using Image-Pro Plus 6.0 software, and the average optical density value of the three fields was recorded.

#### 2.4.3 Western blotting

Five cryopreserved acupoint tissue samples (approximately 100 mg each) were taken from each group. The tissue samples were lysed and homogenized with RIPA lysis buffer, then fully lysed on ice for 30 min. The lysate was centrifuged at 12,000 rpm for 5 min at 4°C, and the supernatant was collected. Protein concentration was determined using the BCA method (A G3422, B G3522, GBCBIO Technologies Inc.). The samples were denatured by boiling for 10 min, followed by protein loading, electrophoresis, membrane transfer, and blocking. The membranes were incubated overnight at 4°C with primary antibodies: rabbit polyclonal anti-TRPV2 (1:1,000) (Bs-10297R, Bioss, USA), rabbit polyclonal anti-TRPV4 (1:2,000) (DF8624, Affinity, USA), and rabbit polyclonal anti-GAPDH (1:2,000) (AB-P-R001, Hangzhou Goodhere Biotechnology Co., Ltd). The next day, the PVDF membranes were washed five times with TBST (5 min each), incubated with HRP-labeled goat anti-rabbit IgG secondary antibody (1:10,000) (BA1054, Wuhan Boster Biological Technology, Co. Ltd.) at room temperature for 2 h on a shaker, and washed again five times with TBST (5 min each). ECL reagent was applied, and the membranes were exposed in a dark room. The film was scanned, and gray value analysis was performed using Image-Pro Plus 6.0 software. The relative expression level of the target protein was determined by the ratio of the grayscale value of the target band to the grayscale value of the internal control.

#### 2.4.4 Real-time quantitative PCR

Five cryopreserved acupoint tissue samples (approximately 100 mg each) were taken from each group. Total RNA was extracted using the Trizol method (15596–026, Ambion, USA). The purity and concentration of RNA were calculated. cDNA was synthesized by reverse transcription following the kit instructions (R233-01, Nanjing Vazyme), with reaction conditions set at 50°C for 15 min, 85°C for 5 s, and 4°C for 10 min. PCR amplification was conducted with a reaction system totaling 20 μL, including 4 μL of cDNA, 10 μL of SYBR Green Master Mix, 0.4 μL of forward primer, 0.4 μL of reverse primer, 0.4 μL of 50× ROX Reference Dye 2, and 4.8 μL of H_2_O. The amplification conditions were as follows: initial denaturation at 95°C for 10 min, denaturation at 95°C for 15 s, and annealing and extension at 60°C for 60 s, for a total of 40 cycles. Melting curve data was collected under the following conditions: 95°C for 15 s, 60°C for 60 s, and 95°C for 15 s. Using *β*-actin as an internal control, the relative mRNA expression levels were analyzed by the 2-ΔΔCt method, with each sample analyzed in triplicate. Primer sequences are listed in Table 1.

**Table 1:** Primer sequences.

Gene		Primer Sequence (5′-3′)	Product length/bp
Rat b-actin	Forward	TGACGTTGACATCCGTAAAGACC	117 bp
	Reverse	GTGCTAGGAGCCAGGGCAGTAA	
Rat TRPV2	Forward	CCGAAAGTTTACTGAGTGGTGTT	217 bp
	Reverse	GCAGGCGAAGTTGAAGAAGAA	
Rat TRPV4	Forward	CAAGTGGCGTAAGTTCGG	131 bp
	Reverse	TGGTACGGTAAGGGTAGGG	

### 2.5 Statistical analysis

SPSS 23.0 statistical software was used for data analysis, and GraphPad Prism 9.0 was used for statistical charting. Measurement data were expressed as mean ± standard deviation (*x̄*±s). Intra-group comparisons (i.e., comparisons within the same experimental group at different time points) and inter-group comparisons (i.e., comparisons across different experimental groups at the same time point) were performed using one-way analysis of variance (one-way ANOVA). When variances were equal, the LSD method was used for pairwise comparisons; when variances were unequal, the Dunnett T3 method was used. A *p*-value of <0.05 was considered statistically significant. Pearson’s correlation coefficient was used for correlation analysis, with a p-value of <0.05 indicating a significant correlation. A correlation coefficient of 0 < r < 1 indicated a positive correlation, while −1 < r < 0 indicated a negative correlation.

## 3 Results

### 3.1 Comparison of Ca^2+^ fluorescence intensity in tissue cells of Zusanli (ST36) acupoint area among groups

**Figure 2:**
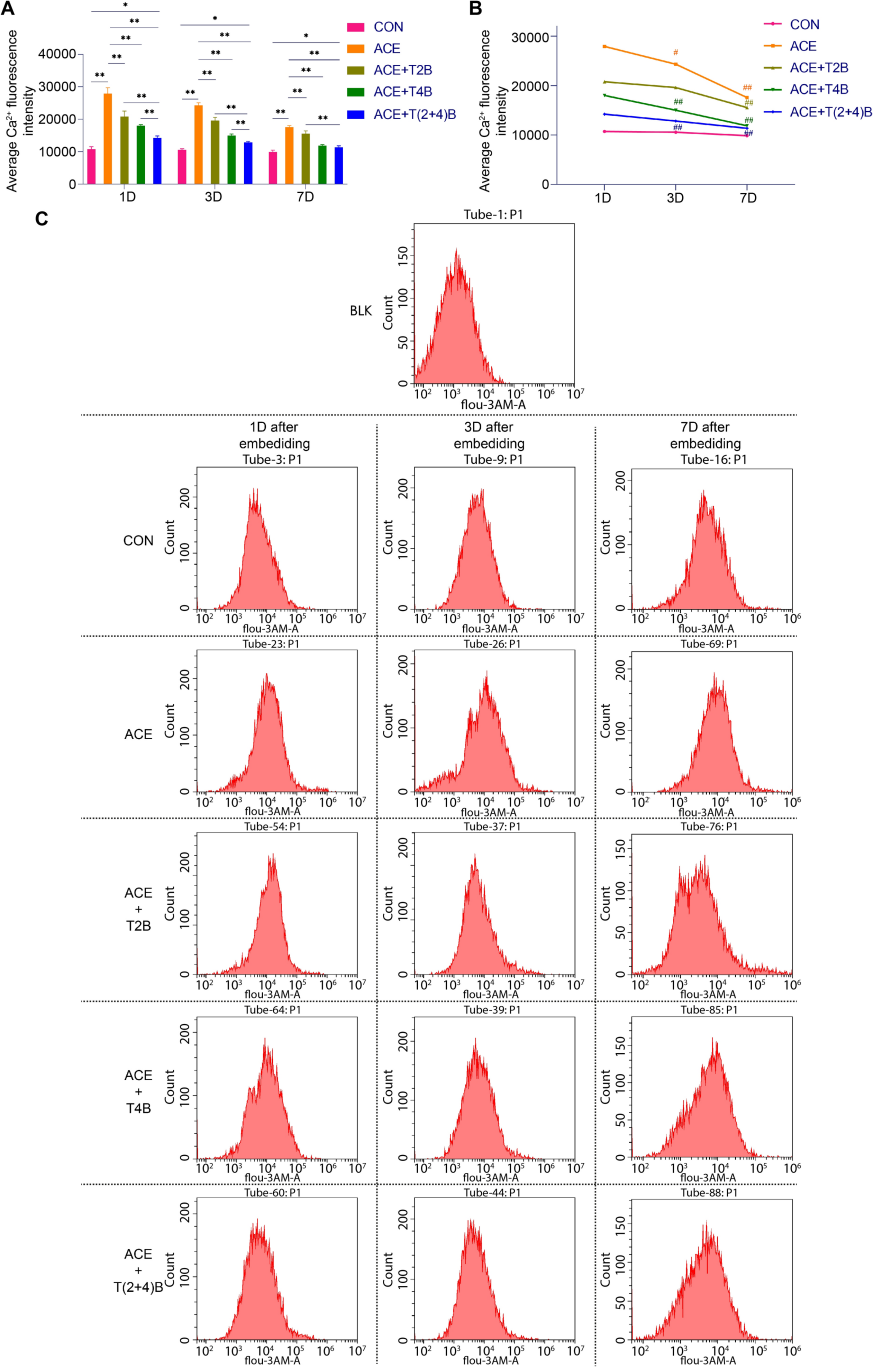
Comparison of Ca^2+^ fluorescence intensity in tissue cells of Zusanli (ST36) acupoint area among groups. **(A)**. Inter-group comparison of intracellular Ca^2+^ fluorescence intensity in tissues of acupoint area at the same time point (*n* = 5 per group). **(B)** Intra-group comparison of intracellular Ca^2+^ fluorescence intensity in tissues of acupoint area across different time points (*n* = 5 per group). **(C)** Detection of intracellular Ca^2+^ fluorescence intensity in tissues of acupoint area among groups by flow cytometry. **(A)** Comparison between groups at the same time point, *p < 0.05, **p < 0.01; **(B)** Comparison of the same group 1 day post-embedding, #*p* < 0.05, ##*p* < 0.01.

In this study, flow cytometry was used to detect the Ca^2+^ fluorescence intensity in the acupoint tissue cell suspension to observe changes in the Ca^2+^ concentration in the local tissue cells following the embedding of PGLA suture in the acupoint and the use of TRPV2 and TRPV4 inhibitors. Compared to the CON Group, the Ca^2+^ fluorescence intensity in the tissue cells of the acupoint area in the ACE Group significantly increased at 1 day, 3 days, and 7 days after embedding. However, compared to the ACE Group, the Ca^2+^ fluorescence intensity in the tissue cells of the acupoint area significantly decreased in the (ACE+T2B) Group, (ACE+T4B) Group, and (ACE+T(2 + 4)B) Group at 1 day, 3 days, and 7 days post-embedding. Additionally, compared to the (ACE+T(2 + 4)B) Group, the Ca^2+^ fluorescence intensity in the tissue cells increased in the (ACE+T2B) Group at 1 day, 3 days, and 7 days post-embedding and increased in the (ACE+T4B) Group at 1 day and 3 days post-embedding (Figures 2A,C). The Ca^2+^ fluorescence intensity in the tissue cells of the acupoint area in all intervention groups showed a decreasing trend over time. Compared to 1 day post-embedding, the Ca^2+^ fluorescence intensity in the tissue cells of the acupoint area significantly decreased at 3 days post-embedding in the ACE Group, (ACE+T4B) Group, and (ACE+T(2 + 4)B) Group, and at 7 days post-embedding in the (ACE+T2B) Group (Figures 2B,C). These results indicate that embedding PGLA suture in the acupoint can affect the Ca^2+^ fluorescence intensity in tissue cells by regulating TRPV2 and TRPV4, and the Ca^2+^ fluorescence intensity gradually weakens over time.

### 3.2 Comparison of positive expression of Mac CD68 and MC tryptase in acupoint tissues among groups

CD68 and tryptase are commonly used markers for Macs and MCs, respectively (Payne and Kam, 2004; Chistiakov et al., 2017). To observe the effect of embedding PGLA suture in acupoints and the use of TRPV2 and TRPV4 inhibitors on the function of these two immune cells, immunofluorescence staining was used to detect the expression of CD68 in Macs and tryptase in MCs in the local tissue of the acupoint area. The results are as follows.

**Figure 3:**
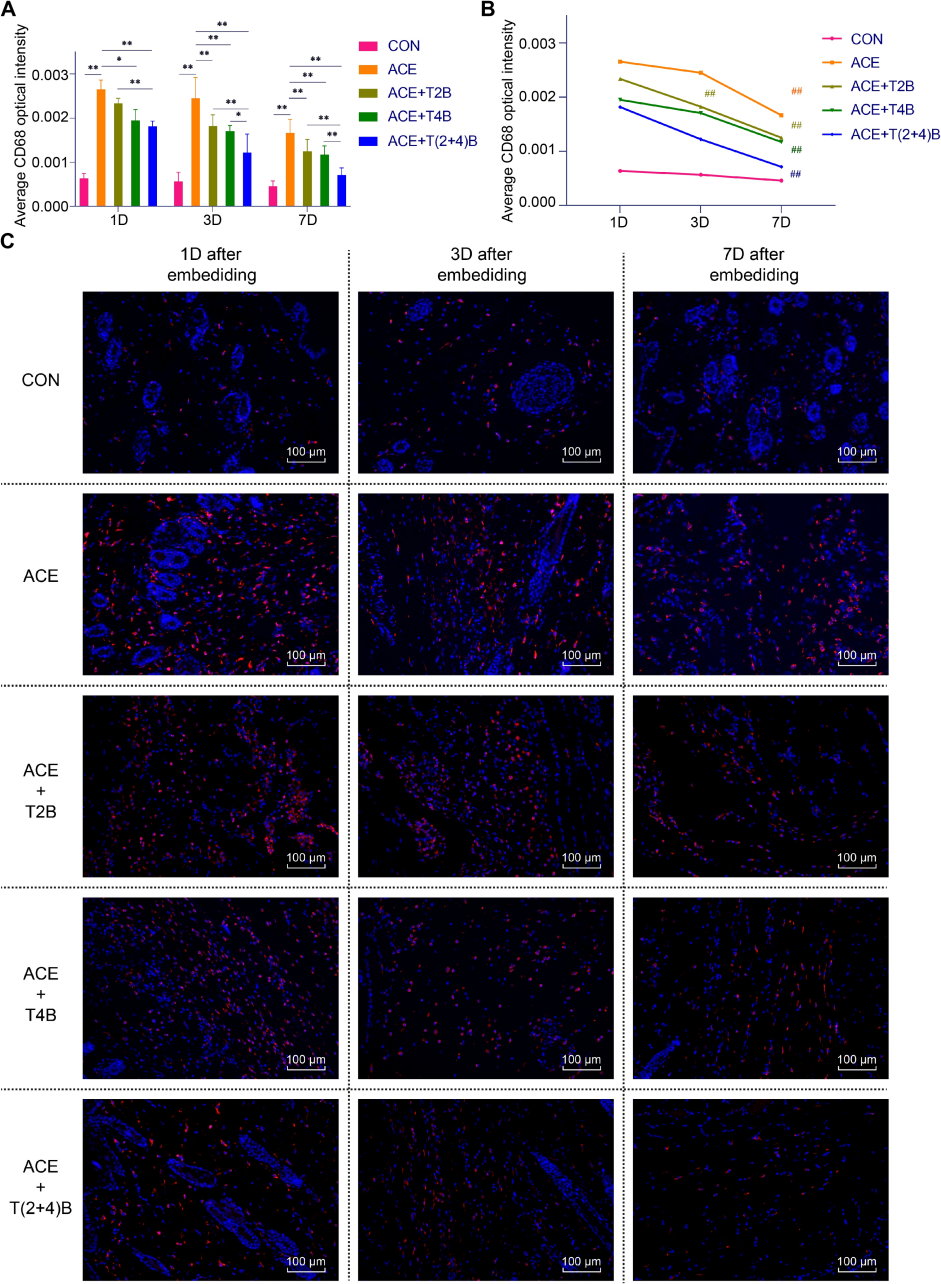
Comparison of positive CD68 expression in Macs in acupoint area tissues: inter- and intra-group analysis. **(A)** Inter-group comparison of positive CD68 expression in Macs in tissues of acupoint area at the same time point (*n* = 5 per group). **(B)** Intra-group comparison CD68 expression in Macs in tissues of acupoint area across different time points (*n* = 5 per group). **(C)** Immunofluorescence staining of CD68 expression in Macs in tissues of acupoint area of each group. **(A)** Comparison between groups at the same time point, *p < 0.05, **p < 0.01. **(B)** Comparison of the same group 1 day after embedding, #*p* < 0.05, ##*p* < 0.01. **(C)** Blue indicates DAPI-stained nuclei, red indicates CD68 positive expression, scale bar = 100 μm.

Compared to the blank control group, the expression of CD68 in Macs in the acupoint area of rats in the embedding group significantly increased at 1 day, 3 days, and 7 days after embedding. However, compared to the embedding group, the expression of CD68 in Macs in the acupoint area of rats in the embedding + TRPV4 inhibitor group and the (ACE+T(2 + 4)B) Group significantly decreased at 1 day and 7 days after embedding. The embedding + TRPV2 inhibitor group showed a significant decrease in CD68 expression at 3 days and 7 days after embedding. Furthermore, compared to the (ACE+T(2 + 4)B) Group, the CD68 expression in Macs increased in the embedding + TRPV2 inhibitor group at 1 day, 3 days, and 7 days after embedding, and increased in the (ACE+T4B) Group at 3 days and 7 days after embedding (Figures 3A,C). The expression of CD68 in Macs in the acupoint area of each intervention group showed a decreasing trend over time. Compared to 1 day after embedding, the CD68 expression in Macs in the embedding group, the (ACE+T4B) Group, and the (ACE+T(2 + 4)B) Group significantly decreased at 7 days after embedding, while in the (ACE+T2B) Group, the CD68 expression significantly decreased at 3 days after embedding (Figures 3B,C).

**Figure 4:**
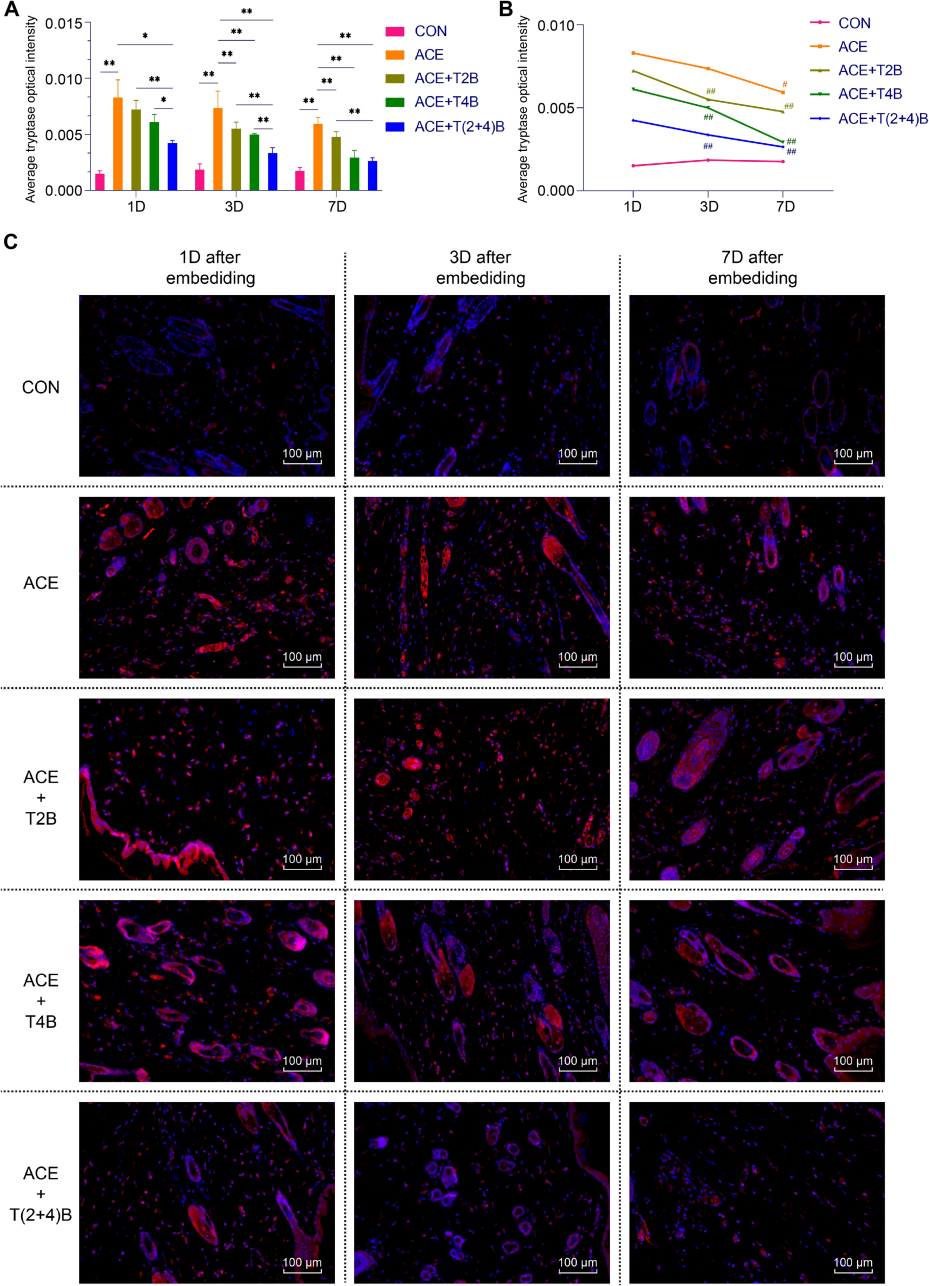
Comparison of positive tryptase expression in MCs in acupoint area tissues: inter- and intra-group analysis. **(A)** Inter-group comparison of positive tryptase expression in MCs in in tissues of acupoint area at the same time point (*n* = 5 per group). **(B)** Intra-group comparison of positive tryptase expression in MCs in tissues of acupoint area across different time points (*n* = 5 per group). **(C)** Immunofluorescence staining of tryptase expression in MCs in tissues of acupoint area of each group. **(A)** Comparison between groups at the same time point, *p < 0.05, **p < 0.01. **(B)** Comparison of the same group 1 day after embedding, #*p* < 0.05, ##*p* < 0.01. **(C)** Blue indicates DAPI-stained nuclei, red indicates tryptase positive expression, scale bar = 100 μm.

Compared to the blank control group, the expression of tryptase in MCs in the acupoint area of rats in the embedding group significantly increased at 1 day, 3 days, and 7 days after embedding. However, compared to the embedding group, the expression of tryptase in MCs in the (ACE+T(2 + 4)B) Group significantly decreased at 1 day, 3 days, and 7 days after embedding. The expression of tryptase in MCs in the (ACE+T2B) Group and the (ACE+T4B) Group significantly decreased at 3 days and 7 days after embedding. Furthermore, compared to the (ACE+T(2 + 4)B) Group, the expression of tryptase in MCs increased in the (ACE+T2B) Group at 1 day, 3 days, and 7 days after embedding, and increased in the (ACE+T4B) Group at 1 day and 3 days after embedding (Figures 4A,C). The expression of tryptase in MCs in the acupoint area of each intervention group showed a decreasing trend over time. Compared to 1 day after embedding, the expression of tryptase in MCs in the (ACE+T2B) Group, the (ACE+T4B) Group, and the (ACE+T(2 + 4)B) Group significantly decreased at 3 days after embedding, while in the embedding group, the expression of tryptase in MCs significantly decreased at 7 days after embedding (Figures 4B,C).

The above results suggest that embedding PGLA suture in acupoints may influenc the expression of CD68 in Macs and tryptase in MCs through TRPV2 and TRPV4, and the expression levels gradually decrease over time.

### 3.3 Comparison of TRPV2 and TRPV4 protein expression in acupoint tissues among groups

In this study, Western blots (WB) were used to verify the regulatory effects of PGLA embedding on local TRPV2 and TRPV4 channels by detecting the expression levels of TRPV2 and TRPV4 proteins in acupoint tissues. The results are as follows.

**Figure 5:**
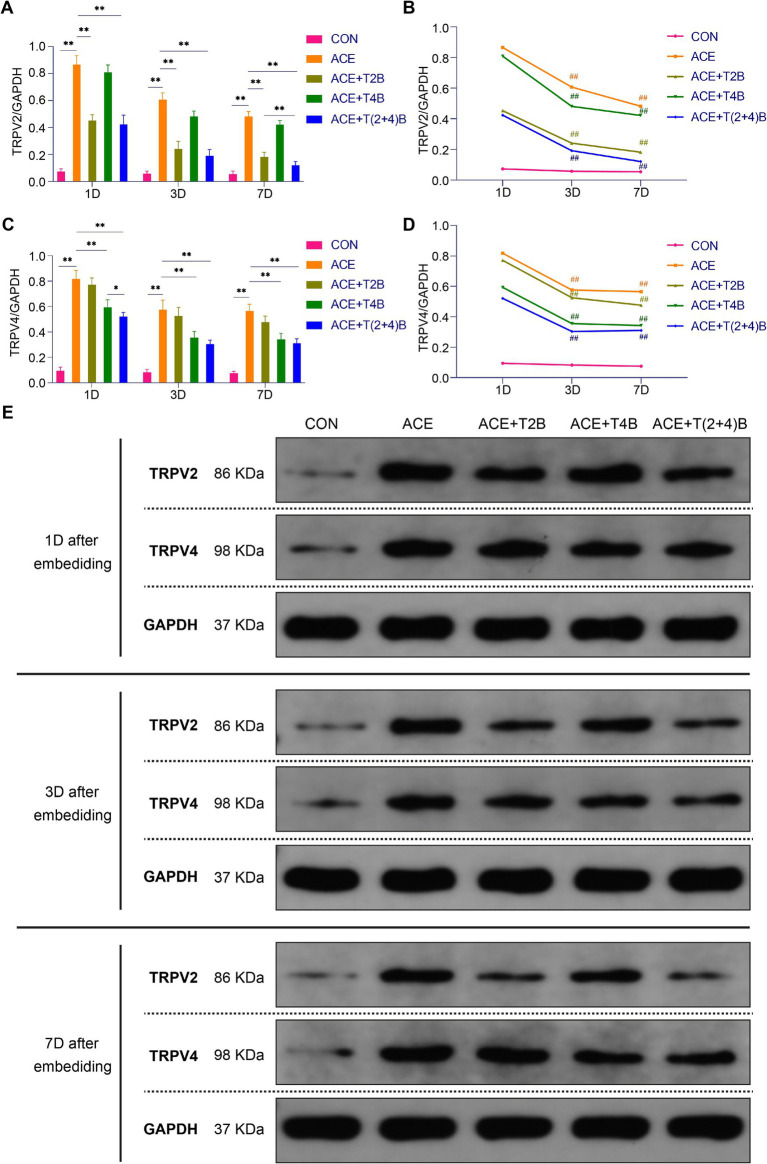
Comparison of TRPV2 and TRPV4 Protein Expression in Acupoint Tissues among Groups. **(A)** Inter-group comparison of TRPV2 protein expression in tissues of acupoint area at the same time point (*n* = 5 per group). **(B)** Intra-group comparison of TRPV2 protein expression in tissues of acupoint area across different time points (*n* = 5 per group). **(C)** Inter-group comparison of TRPV4 protein expression in tissues of acupoint area at the same time point (*n* = 5 per group). **(D)** Intra-group comparison of TRPV4 protein expression in tissues of acupoint area across different time points (*n* = 5 per group). **(E)** TRPV2 and TRPV4 protein expression in tissues of acupoint area of each group of rats. **(A)** Comparison between groups at the same time point, *p < 0.05, **p < 0.01; **(B)** Comparison of the same group 1 day after embedding, #*p* < 0.05, ##*p* < 0.01. **(C)** Comparison between groups at the same time point, *p < 0.05, **p < 0.01. **(D)** Comparison of the same group 1 day after embedding, #*p* < 0.05, ##*p* < 0.01.

Compared to the blank control group, the expression of TRPV2 protein in the acupoint area of rats in the embedding group significantly increased at 1 day, 3 days, and 7 days after embedding. However, compared to the embedding group, the expression of TRPV2 protein in the acupoint area of rats in the (ACE+T2B) Group and the (ACE+T(2 + 4)B) Group significantly decreased at 1 day, 3 days, and 7 days after embedding. Moreover, compared to the (ACE+T(2 + 4)B) Group at the same time point, the TRPV2 protein expression in the (ACE+T2B) Group increased at 7 days after embedding, with no significant difference at 1 day and 3 days after embedding (Figures 5A,E). The TRPV2 protein expression in the acupoint tissue of each intervention group showed a decreasing trend over time. Compared to 1 day after embedding, the TRPV2 protein expression in the embedding group, the (ACE+T2B) Group, the (ACE+T4B) Group, and the (ACE+T(2 + 4)B) Group significantly decreased at 3 days after embedding (Figures 5B,E).

Compared to the blank control group, the TRPV4 protein expression in the acupoint area of rats in the embedding group significantly increased at 1 day, 3 days, and 7 days after embedding. However, compared to the embedding group, the TRPV4 protein expression in the acupoint area of rats in the (ACE+T4B) Group and the (ACE+T(2 + 4)B) Group significantly decreased at 1 day, 3 days, and 7 days after embedding. Moreover, compared to the (ACE+T(2 + 4)B) Group, TRPV4 protein expression in the (ACE+T4B) Group increased at 1 day after embedding, with no significant difference at 3 days and 7 days after embedding (Figures 5C,E). The TRPV4 protein expression in the acupoint area of each intervention group showed a decreasing trend over time. Compared to 1 day after embedding. The TRPV4 protein expression in the embedding group, the (ACE+T2B) Group, the (ACE+T4B) Group, and the (ACE+T(2 + 4)B) Group significantly decreased at 3 days after embedding (Figures 5D,E).

These results indicate that the stimulation formed by embedding PGLA suture in acupoints can regulate TRPV2 and TRPV4 protein expression, which gradually weakens over time.

### 3.4 Comparison of TRPV2 and TRPV4 mRNA expression in acupoint tissues among groups

In this study, the expression levels of TRPV2 and TRPV4 mRNA in the tissues of the acupoint area were simultaneously detected using quantitative fluorescence PCR, to validate the results obtained from Western blot (WB) analysis. The results are as follows.

**Figure 6:**
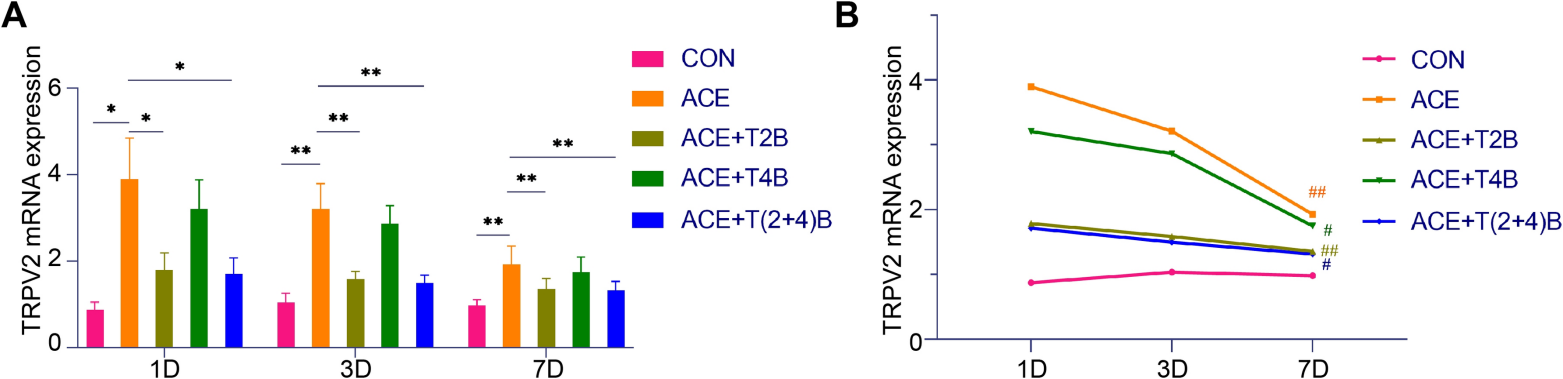
Comparison of TRPV2 mRNA Expression in Acupoint Tissues: inter- and intra-group analysis. **(A)** Inter-group comparison of TRPV2 mRNA expression in tissues of acupoint area at the same time point (*n* = 5 per group). **(B)** Intra-group comparison of TRPV2 mRNA expression in tissues of acupoint area across different time points (*n* = 5 per group). **(A)** Comparison between groups at the same time point, *p < 0.05, **p < 0.01. **(B)** Comparison of the same group 1 day after embedding, #*p* < 0.05, ##*p* < 0.01.

Compared to the blank control group, the expression of TRPV2 mRNA in the acupoint area of rats in the embedding group significantly increased at 1 day, 3 days, and 7 days after embedding. However, compared to the embedding group, the expression of TRPV2 mRNA in the acupoint area of rats in the (ACE+T2B) Group and the (ACE+T(2 + 4)B) Group significantly decreased at 1 day, 3 days, and 7 days after embedding. Moreover, compared to the (ACE+T(2 + 4)B) Group, there was no significant difference in the TRPV2 mRNA expression in the acupoint area of rats in the (ACE+T2B) Group at 1 day, 3 days, and 7 days after embedding (Figure 6A). The expression of TRPV2 mRNA in the acupoint area of each intervention group showed a decreasing trend over time. Compared to 1 day after embedding, the TRPV2 mRNA expression in the embedding group, the (ACE+T2B) Group, the (ACE+T4B) Group, and the (ACE+T(2 + 4)B) Group significantly decreased at 7 days after embedding (Figure 6B).

**Figure 7:**
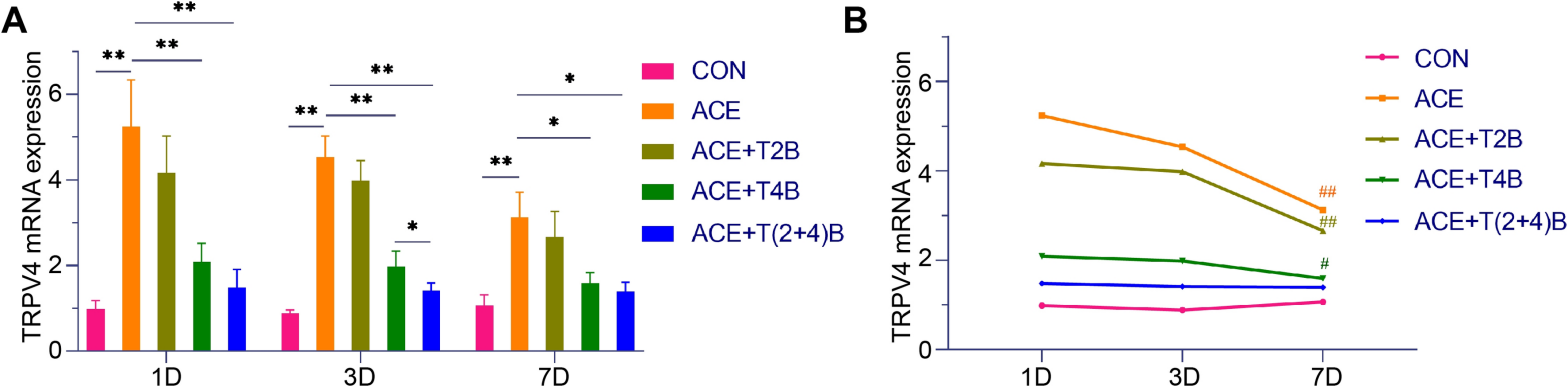
Comparison of TRPV4 mRNA Expression in Acupoint Tissues: inter- and intra-group analysis. **(A)** Inter-group comparison of TRPV4 mRNA expression in tissues of acupoint area at the same time point (*n* = 5 per group). **(B)** Intra-group comparison of TRPV4 mRNA expression in tissues of acupoint area across different time points (*n* = 5 per group). **(A)** Comparison between groups at the same time point, *p < 0.05, **p < 0.01; **(B)** Comparison of the same group 1 day after embedding, #*p* < 0.05, ##*p* < 0.01.

Compared to the blank control group, the expression of TRPV4 mRNA in the acupoint area of rats in the embedding group significantly increased at 1 day, 3 days, and 7 days after embedding. However, compared to the embedding group, the expression of TRPV4 mRNA in the acupoint area of rats in the (ACE+T4B) Group and the (ACE+T(2 + 4)B) Group significantly decreased at 1 day, 3 days, and 7 days after embedding. Moreover, compared to the (ACE+T(2 + 4)B) Group, the TRPV4 mRNA expression in the acupoint area of rats in the (ACE+T4B) Group increased at 3 days after embedding, with no significant difference at 1 day and 7 days after embedding (Figure 7A). The TRPV4 mRNA expression in the acupoint area of each intervention group showed a decreasing trend over time. Compared to 1 day after embedding, the TRPV4 mRNA expression in the embedding group, the (ACE+T2B) Group, and the (ACE+T4B) Group significantly decreased at 7 days after embedding (see Figure 7B).

These results indicate that the local stimulation formed by embedding PGLA suture in acupoints can regulate TRPV2 and TRPV4 mRNA expression, which gradually weakens over time.

### 3.5 Correlation analysis of Ca^2+^ fluorescence intensity and expression of Mac CD68 and MC tryptase in acupoint tissues among groups

**Figure 8:**
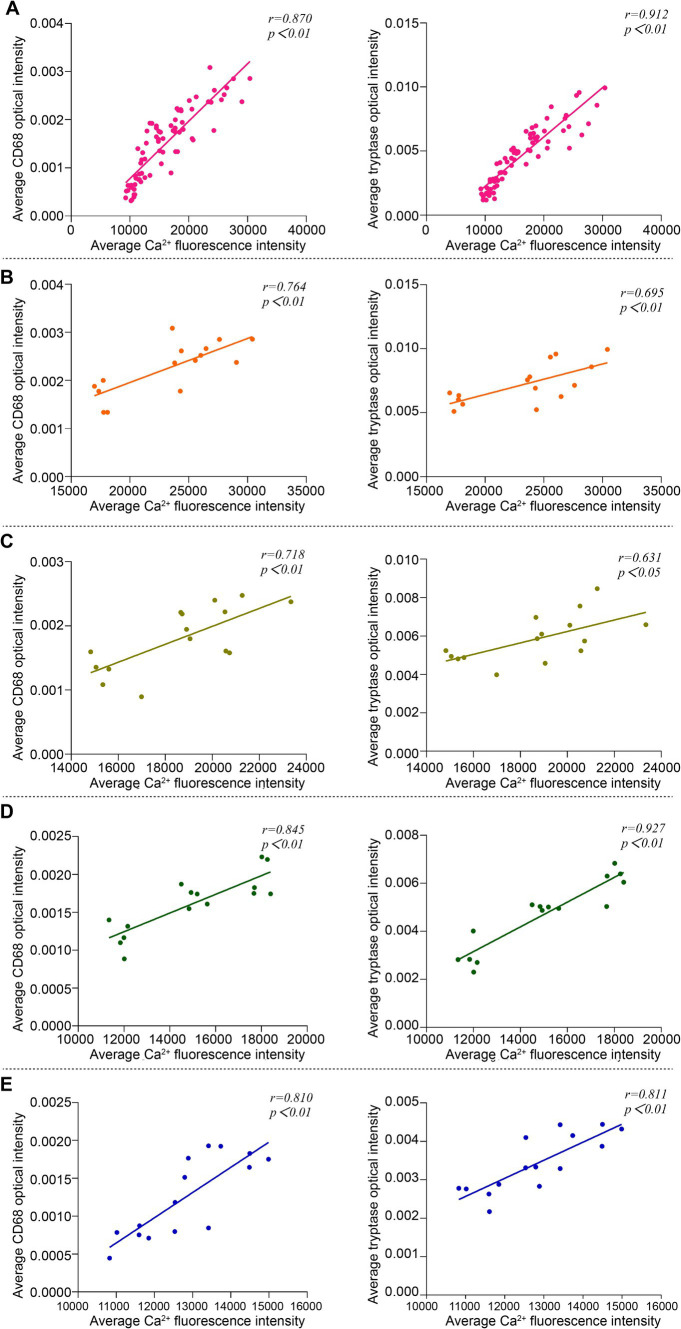
Correlation analysis between Ca^2+^ fluorescence intensity and the expression of Mac CD68 and MC tryptase in acupoint tissues in the overall groups (including the blank control group and the intervention groups) and each intervention group. **(A)** Overall Analysis. **(B)** Embedding group. **(C)** (ACE+T2B) Group. **(D)** (ACE+T4B) Group. **(E)** (ACE+T(2 + 4)B) Group.

Pearson correlation analysis was used to compare the correlation between Ca^2+^ fluorescence intensity and the expression of Mac CD68 and MC tryptase in acupoint tissues in the analysis of the overall groups (including the blank control group and the intervention groups) and individual intervention group. The results showed that in the overall analysis, there was a positive correlation between Ca^2+^ fluorescence intensity and the expression of Mac CD68 and MC tryptase in acupoint tissues (Figure 8A). Analysis of the embedding group, the (ACE+T2B) Group, the (ACE+T4B) Group, and the (ACE+T(2 + 4)B) Group showed a positive correlation between Ca^2+^ fluorescence intensity and the expression of Mac CD68 and MC tryptase in acupoint tissues (Figures 8B–E).

## 4 Discussion

In traditional Chinese medicine theory, the acupoint “Zusanli” (ST36) belongs to the Stomach Meridian of Foot-Yangming. It serves both as the He-Sea point of the Stomach Meridian and the lower He-Sea point of the Stomach Fu organ. Stimulation of this acupoint can harmonize the spleen and stomach, invigorate Qi, and enhance the body’s resistance to diseases, making it widely used in the treatment of various ailments throughout history (Shi, 2017; Chen X. L. et al., 2016). Recent meta-analyses have shown that the application of this acupoint has expanded to include treatment for pain, speech disorders, emotional problems, cognitive impairment, gastrointestinal tumors, adverse reactions to radiotherapy, and postoperative ileus (Huang et al., 2022; Zhou et al., 2020; Wu et al., 2020; Li et al., 2022; Wang et al., 2015). Literature reviews indicate that ST36 is the most frequently used acupoint in both clinical acupuncture studies and basic experimental research (Zhu, 2021; Yang et al., 2018). Based on its extensive use and recognition in research, as well as previous findings from our research team showing inflammatory responses in the local acupoint area of both humans and rats after ACE at ST36 (Zhang X. H. et al., 2023; Zhang Q. et al., 2023; Wang et al., 2023; Liang et al., 2019), this acupoint was used in this experimental study. This study selected 1 day, 3 days, and 7 days post PGLA embedding as observation time points. This choice was mainly based on the regulations set forth by the National Standard of the People’s Republic of China (GB/T 21709.10–2008), which stipulates that the interval between ACE treatments should be at least 1 week (Guan et al., 2009). Additionally, bibliometric studies have indicated that the most commonly adopted interval in clinical practice is 7 days (Cheng et al., 2022a). Furthermore, observations made by the research team using MRI in clinical studies revealed that local stimulation effects after PGLA embedding change significantly within 1 week, typically appearing within 1 day and lasting for 3 to 7 days (Liang et al., 2016). Therefore, this study employed the aforementioned three time points for observation.

Compared to traditional acupuncture, ACE not only involves transient needling but also provides continuous stimulation of the acupoint due to the embedded threads (Wu et al., 2019), which is crucial. Clinically used threads include catgut, polydioxanone (PDO), polydioxanone suture (PDS), chitosan, polyglycolic acid (PGA), and poly(glycolide-co-lactide) (PGLA) (Cheng et al., 2022a). Catgut has long been the dominant material for ACE, but its propensity to cause allergic reactions and related adverse effects due to its foreign protein nature has limited its use, leading to a gradual replacement by newer materials (Ma et al., 2019). Threads like PDS and chitosan are less commonly used in ACE research due to their later emergence, longer degradation times, unclear degradation mechanisms, and higher costs (Ke et al., 2020; Cheng et al., 2022a; Du and Zhang, 2019). Among these materials, PGA and PGLA are derived from natural plants and do not contain animal-derived proteins, offering good biocompatibility. They degrade through hydrolysis in body fluids into carbon dioxide and water, which are excreted from the body (Bajaj and Singhal, 2011; Makadia and Siegel, 2011). Compared to PGA, PGLA has better biodegradability, and foundational research has further proven that PGLA sutures, due to their superior mechanical and hydrophilic properties, are more suitable for use as embedding materials in acupuncture (Jain, 2000; Xu, 2018).

Acupuncture, as an external mechanical and physical stimulus, can activate two types of mechanosensitive TRPV channels expressed on different cell membranes, causing Ca^2+^ influx and signal transduction (Liedtke and Kim, 2005; Luo et al., 2022). ACE, derived from the traditional acupuncture technique of “needle retention,” induces local immune-inflammatory responses at the acupoint due to the foreign body nature of the suture (Zhang X. H. et al., 2023; Zhang Q. et al., 2023; Wang et al., 2023). To verify the mechanical and physical stimulation formed by suture implantation at the acupoint, this study used the inhibitors SKF96365 (Guo Y. Y. et al., 2022) and GSK2193874 (Lawhorn et al., 2021) of the mechanosensitive channels TRPV2 and TRPV4. The results showed that, after embedding PGLA suture, the local tissues of the ST36 acupoint in rats showed an increase in the expression of mRNA and protein in TRPV2 and TRPV4, as well as an increase in intracellular Ca^2+^ fluorescence intensity, both of which decreased over time. When the inhibitors were used, the mRNA and protein expression of TRPV2 and TRPV4 in the local tissues, as well as intracellular Ca^2+^ fluorescence intensity, decreased correspondingly, especially when both inhibitors were used together, resulting in a further reduction in intracellular Ca^2+^ fluorescence intensity compared to using a single inhibitor. These results suggest that the stimulation generated by ACE at the acupoint can modulate the expression of the two mechanosensitive TRPVs (TRPV2 and TRPV4), affecting Ca^2+^ influx in tissue cells, and the stimulation gradually weakens over time. Studies have shown that absorbable sutures gradually soften and are absorbed over time after surgical suturing and embedding at acupoints, leading to a decline in their mechanical properties (Müller et al., 2016; Liang et al., 2019). In light of these findings, the results of this study indicates that the mechanical and physical stimulation at the acupoint post-PGLA embedding may result from the compression and friction of the suture against the local tissue, which diminishes as the suture softens and absorbs within the body.

Macs and MCs are important immune-inflammatory cells in the body, playing crucial roles in regulating inflammatory responses, immune surveillance, and tissue repair (Essandoh et al., 2016; Velez et al., 2018; Xu and Shi, 2012; Ribatti, 2013). They are widely recognized as key participants in initiating the local stimulation effects of acupuncture points (Fu et al., 2023; Dou et al., 2022). In immune-inflammatory responses triggered by foreign bodies, the involvement of these cells differs: Macs primarily clear foreign bodies through phagocytosis and decomposition, whereas MCs mainly trigger inflammatory responses by releasing inflammatory mediators (Velez et al., 2018; Franz et al., 2011). CD68 is a highly glycosylated glycoprotein. Although it is also expressed in other cells, such as synovial cells and neutrophils (Kunisch et al., 2004; Wu et al., 2022), its high expression in Macs makes it a widely used marker for identifying Macs. CD68 can reflect the number and functional activity of Macs in various physiological and pathological processes (Ramprasad et al., 1996; Chistiakov et al., 2017; Seminerio et al., 2018). Our previous study has found that in the ST36 acupoint area of rats, the expression of Mac CD68 in local tissues dynamically changes over time after embedding catgut (Zhang Q. et al., 2023). In this study, embedding PGLA in the same acupoint resulted in elevated CD68 expression in Macs, which gradually weakened over time. Tryptase, a serine protease mainly derived from MCs and released extracellularly upon mast cell degranulation, is the most specific biomarker of mast cell functional activation (Payne and Kam, 2004). Prior research suggests that the degranulation rate of local MCs in the ST36 acupoint of rats moderately changes over time after PGLA embedding (Wang et al., 2023). Correspondingly, in this study, the expression of MC tryptase increased following PGLA embedding in the same acupoint of rats and then showed a gradual decrease over time. The comprehensive results of this experiment reveal that after embedding PGLA in the ST36 acupoint of rats, the expression of CD68 in Macs and tryptase in MCs in the local tissues changes, suggesting that embedding PGLA suture can alter the functions of Macs and MCs in the acupoint area, which gradually diminishes over time. Additionally, in this study, after embedding PGLA in the ST36 acupoint of rats and subsequently injecting TRPV2 and TRPV4 inhibitors, the expression of Mac CD68 and MC tryptase in the acupoint tissue decreased. Notably, when both TRPV2 and TRPV4 inhibitors were used together, the decreasing trend was more significant compared to using a single inhibitor. As previously mentioned, the mechanosensitive TRPV2 and TRPV4 channels (Shibasaki, 2016; Liedtke and Kim, 2005) are expressed on the membranes of both Macs and MCs (Huang et al., 2018; Link et al., 2010; Michalick and Kuebler, 2020; Chen et al., 2017), and CD68 and tryptase are important markers for identifying Macs and MCs, respectively (Chistiakov et al., 2017; Payne and Kam, 2004). Therefore, these changes in research results indicate that the stimulation generated by embedding PGLA suture in the ST36 acupoint can influence the functions of MCs and Macs by modulating mechanosensitive TRPV channels, with the stimulation effects gradually weakening over time.

Ca^2+^ plays an important role as a messenger in signal transduction in tissue cells, a process known as Ca^2+^ signaling, which is a biochemical process (Bootman and Bultynck, 2020). During macrophage functional activation, an increase in intracellular Ca^2+^ concentration can activate a series of downstream signals, promoting phagocytosis (Zhu et al., 2017) and the release of inflammatory factors, cytokines, and enzymes such as tumor necrosis factor-*α* (TNF-α), interleukin-1 (IL-1), interleukin-12 (IL-12), lysosomal enzymes, and matrix metalloproteinases (MMPs) (Kusmartsev et al., 2016; Liu et al., 2016; Chen et al., 2015; Huang et al., 2012), thereby influencing their participation in immune-inflammatory responses. In mast cell degranulation, in addition to Ca^2+^ release from the endoplasmic reticulum, the opening of ion channels on the cell membrane allows Ca^2+^ influx, which serves as a key signal, leading to increased intracellular Ca^2+^ concentration, which promotes cell functional activation and degranulation, as well as the release of bioactive mediators such as tryptase, histamine (HA), and serotonin (5-hydroxytryptamine, 5-HT) (Ma and Beaven, 2011; Tsvilovskyy et al., 2018; Wernersson and Pejler, 2014), thereby mediating immune-inflammatory responses. Thus, changes in intracellular Ca^2+^ concentration, or Ca^2+^ signaling, affect the functions of both Macs and MCs. In this experimental study, the overall correlation analysis suggests that the fluorescence intensity of intracellular Ca^2+^ in acupoint tissues correlates positively with the expression of CD68 in Macs and tryptase in MCs, indicating that changes in intracellular Ca^2+^ concentration affect the functional changes of these two immune cells. Correlation analysis in the embedding group showed that after embedding PGLA in the ST36 acupoint of rats, the fluorescence intensity of intracellular Ca^2+^ in local acupoint tissue cells was positively correlated with the expression of CD68 in Macs and tryptase in MCs. This implies that PGLA embedding influences the functional changes of these two immune cells by regulating intracellular Ca^2+^ concentration in local tissue cells. The regulatory pathway, as verified in this experiment, includes mechanosensitive TRPV channels. However, given that ACE is a complex stimulation therapy, the activation mechanisms of MCs and Macs are intricate (Gilfillan and Tkaczyk, 2006; Tatemoto et al., 2018; Gordon and Martinez, 2010). In addition, in the study results, correlation analysis of the (ACE+T2B) Group, (ACE+T4B) Group, and (ACE+T(2 + 4)B) Group showed that after using the two inhibitors, the correlation coefficient between the fluorescence intensity of intracellular Ca^2+^ in local acupoint tissue cells and the expression of Mac CD68 and MC tryptase did not significantly decrease after embedding PGLA suture, and the fluorescence intensity of intracellular Ca^2+^ in acupoint tissue cells in the embedding + TRPV (2 + 4) group was still higher than that in the blank control group. These results suggest that the regulation of the functions of these two immune cells by Ca^2+^ signaling after embedding PGLA suture in the acupoint is not limited to the two mechanosensitive TRPV channels but involves multiple pathways. Therefore, we propose that embedding PGLA suture in the ST36 acupoint of rats may induce the functional changes of MCs and Macs through Ca^2+^ signaling, which includes coupled participation of the mechanosensitive TRPV channels.

## 5 Conclusion

In summary, embedding PGLA suture in the ST36 acupoint may locally regulate the expression of TRPV2 and TRPV4 through mechanical and physical stimulation, leading to increased intracellular Ca^2+^ concentration in tissue cells. This, in turn couples with Ca^2+^ signaling to affect the functions of MCs and Macs, forming a physico-chemical-immune linkage effect that gradually weakens over time. The findings of this study not only provide new scientific evidence for the local stimulation effects of ACE under normal physiological conditions but also offer important reference points for future exploration of ACE’s role in pathological states involving TRPV2, TRPV4, MCs, and Macs, as well as its distal effects.

However, WB and qPCR results from the experiment suggest that the two inhibitors used may have interactive inhibitory effects on TRPV2 and TRPV4 ion channels, and future research may consider using specific TRPV gene knockout rats for related studies. Additionally, besides TRPV2 and TRPV4, TRPV1 is also expressed in MCs and Macs (Freichel et al., 2012; Zhang et al., 2012; Vašek et al., 2024). TRPV1 is sensitive to mechanical stimulation under specific conditions such as inflammation, tissue injury, and nerve damage (McGaraughty et al., 2008; Vilceanu et al., 2010; Brenneis et al., 2013). Therefore, whether ACE has a stimulatory effect on TRPV1 is also worth further investigation. Moreover, it remains to be verified whether ACE affects other tissue cells at the acupoint (such as neurons, fibroblasts, and endothelial cells) and their factor release through TRPV ion channels (Wu et al., 2015; Fu et al., 2023), and thus creates a cross-tissue effect, which is significant for exploring the potential distal effects of ACE. Research indicates that acupuncture effects are closely related to purinergic signaling, particularly the release of adenosine (ATP, ADP) by MCs triggered by Ca^2+^ influx, which plays a crucial role in acupuncture analgesia (Burnstock, 2009; Müller et al., 2016; Wang et al., 2022). As a complex stimulation therapy developed from acupuncture, whether ACE, in addition to regulating mechanical sensitivity TRPV at the acupoint, can mediate through purinergic signaling to induce Ca^2+^ influx and affect cell function still needs further exploration. Therefore, future studies could delve deeper into the mechanisms of ACE in these aspects.

## Data Availability

The raw data supporting the conclusions of this article will be made available by the authors, without undue reservation.
